# Andean Sprouted Pseudocereals to Produce Healthier Extrudates: Impact in Nutritional and Physicochemical Properties

**DOI:** 10.3390/foods11203259

**Published:** 2022-10-18

**Authors:** Luz María Paucar-Menacho, Marcio Schmiele, Alicia Anais Lavado-Cruz, Anggie Liseth Verona-Ruiz, Carmen Mollá, Elena Peñas, Juana Frias, Wilson Daniel Simpalo-Lopez, Williams Esteward Castillo-Martínez, Cristina Martínez-Villaluenga

**Affiliations:** 1 Departamento de Agroindustria y Agrónoma, Facultad de Ingeniería, Universidad del Santa, Nuevo Chimbote, Ancash 02712, Perú; 2 Institute of Science and Technology, Federal University of Jequitinhonha and Mucuri Valleys, Diamantina 39100-000, Brazil; 3 Department of Food Technology and Biotechnology, Institute of Food Science, Technology and Nutrition (ICTAN), Spanish National Research Council (CSIC), 28040 Madrid, Spain

**Keywords:** pseudocereal grains, germination, extrusion, nutritional quality, bioactive compounds, physicochemical properties, digestion

## Abstract

The tailored formulation of raw materials and the combination of grain germination and extrusion processes could be a promising strategy to achieve the desired goal of developing healthier expanded extrudates without compromising sensory properties. In this study, modifications in the nutritional, bioactive profile and physicochemical properties of corn extrudates as influenced by the complete or partial replacement by sprouted quinoa (*Chenopodium quinoa* Willd) and cañihua (*Chenopodium pallidicaule* Aellen) were investigated. A simplex centroid mixture design was used to study the effects of formulation on nutritional and physicochemical properties of extrudates, and a desirability function was applied to identify the optimal ingredient ratio in flour blends to achieve desired nutritional, texture and color goals. Partial incorporation of sprouted quinoa flour (SQF) and cañihua flour (SCF) in corn grits (CG)-based extrudates increased phytic acid (PA), total soluble phenolic compounds (TSPC), γ-aminobutyric acid (GABA) and oxygen radical antioxidant activity (ORAC) of the extrudates. Sprouted grain flour usually results in an deleterious effect physicochemical properties of extrudates, but the partial mixture of CG with SQF and SCF circumvented the negative effect of germinated flours, improving technological properties, favoring the expansion index and bulk density and increasing water solubility. Two optimal formulations were identified: 0% CG, 14% SQF and 86% SCF (OPM1) and 24% CG, 17% SQF and 59% SCF (OPM2). The optimized extrudates showed a reduced amount of starch and remarkably higher content of total dietary fiber, protein, lipids, ash, PA, TSPC, GABA and ORAC as compared to those in 100% CG extrudates. During digestion, PA, TSPC, GABA and ORAC showed good stability in physiological conditions. Higher antioxidant activity and amounts of bioaccessible TSPC and GABA were found in OPM1 and OPM2 digestates as compared to those in 100% CG extrudates.

## 1. Introduction

The snack food market has continued to grow over the years, particularly in the gluten-free sector, as their availability and convenience attract consumer attention [1,2]. Among the different technologies used for snack production, extrusion is one of the most innovative and interesting processes [1,3]. Extrusion flexibility enables several unit operations such as mixing, heating, shearing, shaping, texturing and drying within one process in a continuous and cost-efficient manner. The process can be applied to a wide range of raw materials to achieve very specific textures, shapes and nutritional properties in food products. Extrusion modifies the structure of raw materials and changes its techno-functional properties by application of pressure, high temperature and shear force [4]. From a nutritional point of view, thermoplastic extrusion is known to offer several advantages that include the development of flavor; inactivation of lipolytic enzymes (thus increasing the shelf-life); the degradation of antinutrients (lectin, tannins, phytic acids, enzyme inhibitors); the increase of soluble dietary fiber; the improvement of starch and protein digestibility and the increase of bioavailability of health-promoting phytochemicals, vitamins and minerals [5]. Therefore, there is great potential for the global food industry to manipulate the nutritional status of extrudates from highly digestible starch and protein products for people indulging in sport activities, to relatively low-glycemic-index and highly bioactive extrudates for those consumers interested in maintaining a balanced nutrition [1].

Corn, potato, rice and wheat are common raw materials for the development of extrudate snacks due to their high starch content and good expansion index (EI) [1]. The resulting products are usually highly dense in energy, with high amounts of easily digestible carbohydrates and low content of proteins and dietary fiber. On the other side, pseudocereal grains are currently trending as they are gluten-free and have excellent nutritional and bioactive profiles [6]. In particular, quinoa (*Chenopodium quinoa* Willd) and cañihua (*Chenopodium pallidicaule* Aellen) are rich in dietary fiber and high-quality protein with a balanced essential amino acid composition [6,7]. They are also a good source of minerals, vitamins and phytochemicals with potential health benefits (polyphenols, phytosterols, phytosteroids and betalains) [6]. Pseudocereal flours have lower starch amounts than those of major cereal grains do, being promising alternatives for the development of food with a lower glycemic load [6]. Along with their beneficial properties, pseudocereals contain several antinutritional compounds, such as saponins and phytates, as well as molecules that may have detrimental effects on the organoleptic properties of the derived foods [8]. Therefore, specific treatments are required to remove these non-nutritive compounds.

Pseudocereal grains and flours are still underutilized raw materials for the production of expanded extrudates [4]. To completely or partially replace common cereals in extruded products, the expansion and mechanical properties of these grain alternatives should be intensively studied and evaluated. Comparative studies on the effect of extrusion on these grains, however, are scarce. Previous works demonstrated that pseudocereal grains could not fully replace common cereals due to their lower sectional expansion index (EI) [9]. Therefore, to obtain products of maximum overall quality, the selection of extrusion parameters and proper feed formulation is still necessary [10].

The use of sprouted grains in the production of expanded extrudates is emerging as a promising opportunity to develop healthier snacks. According to several studies, sprouted cereal grains have higher palatability and increased nutritional quality than ungerminated grains do [11]. Germination activates the hydrolysis of the main nutrients (starch, protein, fiber and lipids) while inducing *de novo* biosynthesis of health-promoting metabolites such as phenolic compounds and γ-aminobutiric acid (GABA) [11,12]. These modifications are often linked to improved nutritional quality of extrudates made of sprouted cereal grains for their higher digestibility, increased amount of vitamins and essential amino acids, lower levels of antinutrients and higher amount of bioactive compounds [13]. The use of sprouted cereal grains additionally improves the physicochemical properties of extrudates. For instance, sprouted wheat extrudates are easier to break, have lower bulk density (BD), increased longitudinal EI and cold-water solubility [14,15]. Moreover, extrusion of sprouted grains improves the sensory properties. Earlier work has shown that sprouted wheat extrudates have increased sweetness that is attributed to starch degradation and subsequent release of reducing sugars in the sprouting process that are further involved in the formation of Maillard reaction products that give the extrudate a brownish color [16].

The aim of this work was to develop healthy snacks through the supplementation of corn with sprouted pseudocereal grains. Pseudocereal sprouts were processed without discharging waste, which is in accordance with the principles of the bioeconomy [17]. An additional objective was to study the effects of varying corn, sprouted quinoa (SQF) and cañihua flour (SCF) formulations on nutritional and physicochemical properties of extrudates. The simplex centroid mixture design and desirability method were used to obtain a tailored formulation for the development of highly bioactive (total soluble phenolics and GABA) extrudates with improved nutritional quality (higher amounts of protein, dietary fiber, minerals, antioxidant activity and lower levels of available carbohydrates and phytic acid) and acceptable physicochemical properties (EI, BD, water absorption index [WAI], water solubility index [WSI] and instrumental color).

## 2. Materials and Methods

### 2.1. Materials

Quinoa (*Chenopodium quinoa* Willd) var. Pasankalla was obtained from Programa de Investigación y Proyección Social de Cereales y Granos Nativos, Universidad Nacional Agraria La Molina (Lima, Perú). Corn grits (GC, *Zea mays* L.) were supplied from the Caserio de Huacacorral (La Libertad, Perú)). Cañihua (*Chenopodium pallidicaule* Aellen) was obtained from the Estación Experimental Agraria, Instituto Nacional de Innovación Agraria (ILLPA-INIA, Puno, Perú). All chemicals were of standard analytical grade and obtained from Sigma-Aldrich (St. Louis, MO, USA).

### 2.2. Quinoa and Cañihua Germination

Quinoa and cañihua grains were washed with tap water and disinfected in 0.1% sodium hypochlorite for 30 min. Grains were washed with tap water to reach neutral pH and soaked in sterile water at a ratio of 1:5 *w*/*v* for 6 h. Germination was performed in a pilot-scale germination chamber (Maquilak, Lima, Peru). Imbibed grains were sprouted at their optimal germination conditions (20 °C for 42 h for quinoa and 20 °C for 72 h for cañihua) to increase the content of bioactive compounds and antioxidant capacity [18,19]. The sprouts were air dried in a semi-industrial tray dryer (Thorr, model SBT-10XL, Peru) with ventilation at 40 °C for 30 h and ground in a laboratory mill (Brabender, Duisburg, Germany). SQF and SCF were stored in vacuum plastic bags at −20 °C.

### 2.3. Pilot-Scale Extrusion-Cooking

Snacks were made on a pilot-scale extruder at the Instituto de Investigación Tecnológico Agro-industrial (Universidad Nacional del Santa, Chimbote, Perú). Different formulations were prepared according to a simplex centroid mixture design including different ratios of CG, SCF and SQF as shown in Table 1. All ingredients were mixed by hand for 2 min to ensure their proper homogenization.

**Figure 1:**
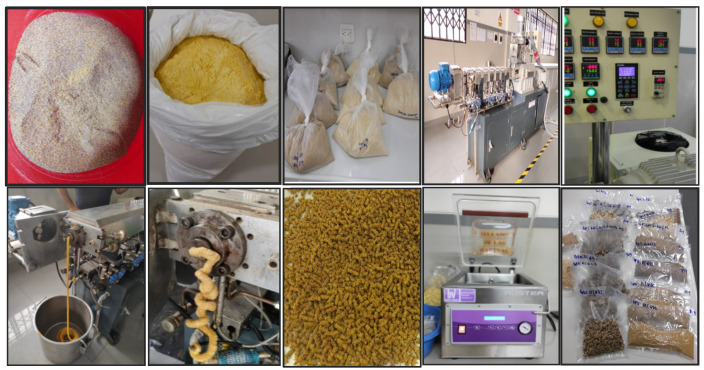
Extruded production workflow. From left to right: At the top—weighing and blending of raw materials; setting up of operation parameters; flour blends feeding and water conditioning. At the bottom—extrusion and cutting of the extrudate into pieces; cooling and storage of the extrudate in bioriented polypropylene bags.

The production workflow of the extrudates is shown in Figure 1. The extrusion process was carried out in a twin-screw extruder (Inbramaq, Labor PQ DRX-50 model, Ribeirão Preto, Brazil) with seven heating zones, a screw length of 870 mm, screw diameter of 32 mm and a die hole diameter of 6 mm. The temperatures in each of the seven zones of the barrel were 30, 45, 55, 75, 95, 105 and 115 °C, respectively, with a screw speed of 304 rpm and feed rate of 6 kg/h. In addition, the flours of the different treatments were previously conditioned at 14% moisture content and kept at refrigerated temperature (~7 °C) before extrusion for water balance. The feeding system consisted of a horizontal twin-screw feeder. The expanded extrudate then came out through the die and was cut with a rotary blade at a speed of 15 Hz. After extrusion, the extrudates were cooled, stored in bioriented polypropylene bags and kept in a dark environment.

### 2.4. Simulated Gastrointestinal Digestion

Simulated gastrointestinal digestion of the extrudates was performed following the INFOGEST 2.0 method [20]. Briefly, 3 g of the extrudates was homogenized in 3 mL of a simulated salivary fluid (pH 7) and incubated in an orbital shaker for 2 min at 37 °C after addition of 75 U/mL of salivary α-amylase (New Brunswick Scientific, Edison, NJ, USA). Afterwards, 5.7 mL of simulated gastric fluid (pH 3) was added, and sample digestion continued with the addition of 0.3 mL of pepsin solution at a final concentration of 2000 U/mL. Digestion tubes were incubated in an orbital shaker for 120 min at 37 °C. Subsequently, pH was adjusted to 7 with 1 M NaOH, and 12 mL of intestinal fluid containing 800 U/mL pancreatin and 10 mM bile was added. Digestion tubes were incubated for 120 min at 37 °C. Intestinal digests were thermally treated for enzyme inactivation at 95 °C for 10 min. The digests were freeze-dried (Virtis Company, Gardiner, NY, USA) and stored at −20 °C.

### 2.5. Nutritional Characterization

Moisture content of raw materials and extrudates was analyzed using the American Association of Cereal Chemists (AACC) method 44–15.02, in which the samples (2.0 g) were dried for 3 h at 130 °C. nitrogen was determined by combustion method using a LECO FP428 nitrogen analyzer (LECO Corporation, St Joseph, MN, USA). Protein content was calculated form nitrogen content (%) using a conversion factor of 5.7. Fat and ash contents were determined using AACC official methods 30–10 and 08–03, respectively [21]. Starch and phytic acid contents were measured using enzymatic kits K-TSTA-100A and K-PHYT (Megazyme Wicklow, Ireland), respectively. The results were expressed in g/100 g of dry weight (dw).

### 2.6. Total Soluble Phenolic Compounds (TSPC)

Total soluble phenolic compounds (TSPC) were analyzed according to Pico et al. [22] with slight modifications [23]. Briefly, 50–100 mg of the milled sample containing 1 mL of 80% methanol in 0.1% formic acid was incubated in a Thermomixer C (Eppendorf AG, Hamburg, Germany) at 30 °C and 2000 rpm for 15 min. After sample centrifugation (Centrifuge 5424 R, Eppendorf AG, Hamburg, Germany) for 5 min at 5 °C and 10,000 rpm, the supernatant was collected. A second cycle of extraction with 1 mL of 70% acetone in 0.1% formic acid was performed following the same protocol. Supernatants of the two cycles of extraction were combined and adjusted to a final volume of 2 mL with distilled water. The extract (1 mL) was mixed with 0.1 mL of 0.1% fast blue BB reagent in distilled water and 0.1 mL of 5% NaOH. The tubes were incubated in the dark at room temperature for 120 min. The samples were placed in a microplate, and absorbance was read at 420 nm using a Synergy HT microplate reader (BioTek Instruments, Winooski, VT, USA). Gallic acid was used as a standard at a concentration range between 2 and 225 µg/mL. The data were expressed as mg of gallic acid equivalents (GAE)/100 g dw.

### 2.7. γ-Aminobutyric Acid (GABA)

Samples (200 mg) were extracted in 2 mL of 0.1 N HCl (allowing the maximum solubilization of proteins, peptides and free (soluble) amino acids) at 5 °C for 30 min using a Thermomixer C (Eppendorf, Madrid, Spain). The samples were centrifuged for 30 min at 5 °C and 8000*× g* (Centrifuge 5424 R, Eppendorf AG, Hamburg, Germany), and supernatants were filtered through 0.22 μm nylon membranes. Analysis of GABA in supernatants was performed by reversed-phase high-performance liquid chromatography (RP-HPLC) and UV detection after pre-column derivatization with 9-fluorenylmethoxycarbonyl chloride (FMOC) and *o-phthaldialdehyde* reagents (Agilent, Santa Clara, CA, USA). Chromatographic separations were carried out in an Agilent 1200 high-performance liquid chromatograph (Agilent, Santa Clara, CA, USA) equipped with a G1314B diode array detector (DAD) and a Zorbax Eclipse Plus C_18_ stationary phase column (4.6 × 150 mm, 3 μm). Mobile phase A was composed of 10 mM Na_2_HPO_4_:10 mM Na_2_B_4_O_7_, pH 8.2: 5 mM NaN_3_, and mobile phase B consisted of acetonitrile–methanol–water (45:45:10, *v*:*v*:*v*). All mobile-phase solvents were of HPLC grade. The analyses were performed at 40 °C, with a flow rate of 1.5 mL/min and the following solvent gradient: 57% B in 20 min, 100% B in 20.1 min, 100% B in 23.5 min, 2% B in 23.6 min and 2% B in 25 min. The DAD detector was set to 338 nm (from 0–15 min) and 262 nm (from 15–30 min). External calibration was carried out using standard solutions of GABA in the linear range between 10 and 1000 nmol/mL (R^2^ > 0.99). The results were expressed as mg/100 g dw.

### 2.8. Antioxidant Activity

Antioxidant activity was determined by the oxygen radical absorbance capacity (ORAC) method as described previously [24]. Briefly, the reaction was performed at 37 °C in 75 mM phosphate buffer at pH 7.4. The reaction mixture (200 μL) contained 180 μL of 70 nM fluorescein, 90 μL of 12 mM 2,2′-azobis(2-amidinopropane) dihydrochloride (AAPH) and 30 μL of a diluted sample or the standard 6-hydroxy-2,5,7,8-tetramethylchroman-2-carboxylic acid (Trolox) at concentrations ranging from 1 to 160 μM. Reaction mixtures were placed in a black 96-well plate, and the fluorescence was read in a Synergy HT microplate reader (BioTek Instruments, Winooski, VT, USA) every minute at excitation and emission wavelengths of 485 and 520 nm, respectively. The equipment was controlled by Gen5™ software, version 1.1 (BioTek Instruments, Winooski, VT, USA). The results were expressed as μmol Trolox equivalents (TE)/100 g dw.

### 2.9. Physicochemical Properties

The EI was calculated between the ratio of the average diameter of the expanded extrudates and the diameter of the die of the extrudates in the expansion zone of the extruder [25].

The BD of the extrudates was determined through the measurements of the dimensions of the samples (length and diameter) using an analog Western caliper (professional model 150 mm), and the weight of the samples was determined on an AUY220 analytical balance (Shimadzu, Tokyo, Japan). The analysis was performed with six replications. The calculation was made through the ratio between the weight and the volume of the sample, as shown in Equation (1) [25].

BD (g/cm^3^) = w/πld^2^ (1)

where w is the sample weight (g), l is the length (cm) and d is the diameter (cm) of the sample.

The instrumental texture of the extrudates as determined by the shear force (SF) and shear work (SW) was analyzed according to AACC method 74–09.01 using a texturometer TA-XT Plus (Micro Systems Stable, Surrey, ENG) equipped with a load cell adjusted to measure the compressive force using a Warner Blatzer knife. The extrudates were placed individually on the platform and processed under the following conditions (probe distance: 90 mm; pre-test speed: 0.5 mm/s; test speed: 0.5 mm/s; and post-test: 10 mm/s). SF (expressed in N) was the first compression required for the sample to break, while SW (expressed in N·s) was the total number of force peaks measured throughout the test. The determination was made with 12 repetitions for each of the 12 formulation tests.

The water absorption index (WAI) and the water solubility index (WSI) were performed according to the Anderson et al. [26] methodology. Both analyses were applied to the flours prior to the extrusion process for the extruded products.

The CIELAB color was determined in triplicate using a colorimeter (Minolta, CR-310, Osaka, Japan). In the CIE color space, L* is lightness, a* is greenness/redness, and b* is blueness/yellowness of extrudates.

### 2.10. Experimental Design and Optimization of Flours Blend Ratio

Flour formulation was optimized using the simplex centroid mixture design including CG (*X_1_*), SQF (*X_2_*) and SCF (*X_3_*) as independent variables. In an experiment with *q* components, the proportions of the ingredients were denoted by *X_1_*, *X_2_*, …, *X_q_*, where *X_i_* ≥ 0 for *i* = 1, 2, …, *q* and ∑*q*_i_ = 1*X_i_* = 1, where x_i_ represents the proportion of the component. This equation removes a degree of freedom from the proportions and the factor space is, therefore, a (*q* − 1)-dimensional regular simplex [27]. The design enabled us to approximate the experimental data (Y_obs_) with a response surface model represented in Equations (2)–(4):

(2) $$\mathrm{Linear}\;\overset{ˇ}{y}=\sum q_{i}=1\beta_{i}X_{i}$$

(3) $$\mathrm{Quadratic}\;\overset{ˇ}{y\;}=\sum q_{i}=1\beta_{i}X_{i}+\sum q-1\sum_{}^{i<j}q_{j}\beta_{ij}X_{i}X_{j}$$

(4) $$\mathrm{Special}\;\mathrm{cubic}\;\overset{ˇ}{y}=\sum q_{i}=1\beta_{i}X_{i}+\sum q-1\sum_{}^{i<j}q_{j}\beta_{ij}X_{i}X_{j}+\sum q-2\sum_{}^{i<j}q-1\sum_{}^{j<k}q_{k}\beta_{ij}kX_{i}X_{j}X_{k}$$

Parameter *β_i_* represents the expected response to the pure blend *X_i_* = 1 and *X_j_* = 0 when *j* ≠ *i*. The term ∑*q*_i_ = 1*β_i_X*_i_ represents the linear blending portion. When curvature arises from nonlinear blending between component pairs, parameters *β_ij_*, which represent either synergistic or antagonistic blending, will be different from zero [28].

The difference between the experimental data (Y_obs_) and model (Y_calc_) gives the relative deviation (ε). For each response, R^2^ (squared correlation coefficient) was calculated, which is the fraction of variation of the response explained by the model. For this work, a coefficient of determination higher than 0.80, significance level < 0.10 and ratio of F_cal_/F_tab_ ≥ 3.0 were adopted to ensure a good prediction of the mathematical models, without lack of fit. The response variables were nutritional parameters (PA, GABA, TSPC and ORAC) and physicochemical variables (EI, BD, WAI, WSI and CIELAB color parameters L*, a* and b*).

The desirability function was used to optimize response variables within a desired range. Design Expert 10 (Stat-Ease Inc., Minneapolis, MN, USA) software was used to assign a level of importance to each response variable and an objective (maximize, minimize or keep in range). All formulations were analyzed during the optimization process, and the two formulations with higher desirability were selected for additional nutritional and physicochemical evaluations.

### 2.11. Statistical Analysis

The data were expressed as the mean ± standard deviation of three independent replicates. One-way analysis of variance (ANOVA, three or more data sets) with a Bonferroni post hoc test was conducted assuming a Gaussian normal distribution and homogeneity of variances. Regression models for PA, GABA, TSPC, ORAC, EI, BD, WAI, WSI and CIELAB color (L*, a*, b*) were generated using Design Expert 10 (Stat-Ease Inc., Minneapolis, USA). ANOVA regression models were created to choose the most significant model (*p* ≤ 0.10) and the best fit (R^2^ > 0.80). Response surfaces and a desirability methodology were used to identify the optimal formulations.

## 3. Results and Discussion

### 3.1. SQF and SCF Had Higher Nutritional and Bioactive Value Than CG

The chemical composition of CG as compared to SQF and SCF is shown in Table 2. CG showed typical nutritional composition values in accordance to previously reported data [29,30]. Even though there was a variation in the nutritional composition of sprouted grains that relied on genotype and germination conditions, the nutritional compositions of SQF and SCF were in accordance with previous studies [31,32]. The comparative analysis of the nutritional profile of SQF and SCF as compared to that of CG denoted clear differences among raw materials (Table 2). Sprouted pseudocereal grains were characterized by significantly lower starch content and remarkably higher total (TDF) insoluble (IDF) and soluble dietary fiber (SDF), protein and fat (*p* ≤ 0.05, Table 2) as compared to those of CG. This is of particular relevance as the Andean region carries a double burden of malnutrition with a high prevalence of cardiometabolic diseases associated, among other causes, with high postprandial blood glucose levels linked to starchy food consumption [33]. A lower starch content may be particularly relevant in cereal-based food formulations to lower their glycemic index (GI) values [34]. A lower GI has a number of effects on the body’s metabolism and physiological functions, including the reduction of insulinemic and glycemic responses to food and protection against insulin resistance and type 2 diabetes [35]. Proteins may display beneficial effects to human health. Protein-rich foods may aid in weight management, providing a sense of fullness and satiety [36]. Dietary fiber plays an important role in human health by reducing constipation, decreasing the risk of noncommunicable diseases such as cardiovascular diseases, colon cancer and type 2 diabetes [37]. In addition, dietary fiber acts as a functional food and is used in weight-control diets due to its low caloric value. Additionally, germination may provide additional nutritional benefits that include improved starch and protein digestibility [31]. Therefore, the incorporation of flours such as sprouted pseudocereal grains in extruded snacks may be a good strategy to improve the nutritional and functional value of food products. IDF was the main fraction in sprouted pseudocereals, representing up to 68% of TDF, in agreement with other studies [27], while SDF was the predominant dietary fiber fraction in CG, accounting for 77% of TDF. Previous work has also highlighted a higher nutritional value of Andean pseudocereal grains (quinoa, cañihua and amaranth) as compared to that of major cereal grains such as wheat, corn and rice [6].

Sprouted pseudocereal flours were a better source of minerals since they showed higher ash content as compared to that of CG (*p* ≤ 0.05, Table 2). PA binds minerals, reducing their bioavailability; therefore, several studies pointed out germination as a useful pretreatment to obtain pseudocereal flours with higher mineral bioavailability and improved protein digestibility [38]. Although quinoa and cañihua PA content was reduced after germination in selected optimal conditions used herein [31], the remaining PA levels were still significantly higher in SQF and SCF as compared to that in CG (*p* ≤ 0.05, Table 2).

Regarding bioactive compounds, SCF and SQF were better sources of GABA and TSPC than CG was (*p* ≤ 0.05, Table 2). Several studies support this finding, as sprouted grains are rich sources of GABA, free phenolic acids and flavonoids as compared to ungerminated grains [11,18,39,40]. Germination is known to increase remarkably TSPC and GABA by activation of seed metabolism [18,40,41]. Specifically, activation of glutamate-decarboxylase (GAD) during seed germination is linked to the conversion of glutamate into GABA [42]. It is also reported that during the germination process, there is an activation of a transferase enzyme that increases glutamic acid subsequently converted to GABA. On the other hand, the activation of cell-wall-degrading enzymes, esterases and phenolic compounds’ biosynthesis routes such as the phenylpropanoid pathway are behind the mechanisms explaining the increased concentrations of soluble polyphenols reported for sprouted grains [11].

Additionally, germinated pseudocereals have shown ORAC values higher than those of ungerminated grains, which has been consistently attributed in the literature to the increased amounts of not only soluble phenolic compounds but also other antioxidant compounds such as vitamins [18,31,41].

Comparing both pseudocereal flours, SCF and SQF showed similar nutritional composition, although SCF was a better source of TDF, TSPC and higher antioxidant activity, while SQF stood out for its higher content of starch, protein and ash (*p* ≤ 0.05, Table 2).

### 3.2. Incorporation of Sprouted Pseudocereal Flours in the Formulation of Extrudates Increased PA, TSPC, GABA and ORAC

There was a significant variation in the PA, TSPC, GABA and ORAC of the extrudates, influenced by formulation (Table 3). The content of PA, GABA, TSPC and ORAC ranged from 0.35 to 0.62 g/100 g, 5.19 to 41.02 mg/100 g, 141 to 1918 mg GAE/100 g and 19.10 to 122.67 μmol TE/g, respectively. As compared to the reference formulation (100% CG), the incorporation of sprouted pseudocereal flour into extrudate formulations slightly increased PA levels (1.7-fold), whereas GABA, TSPC and ORAC reached values up to 7.9, 6.0 and 11.3 times higher, respectively (*p* ≤ 0.05, Table 3). The comparative analysis of all experimental formulations revealed that the use of 100% SCF allowed reaching the highest values in most of the parameters studied (PA, TSPC and ORAC). These results agree with previous studies showing that partial replacement of refined wheat flour by sprouted kiwicha, cañihua and quinoa flours is a good strategy to fortify bread and biscuits in bioactive compounds while improving their antioxidant activity [31,32]. The comparison of PA values in extrudates formulated with 100% CG, SQF and SCF (Tests #1–3, Table 3) and their corresponding flours (Table 2) revealed that the extrusion process did not influence this antinutrient in the operational conditions of this study. There are discrepancies in the literature among the effect of extrusion on PA content as a result of different operational conditions and nutritional composition of the raw materials selected in different studies. Despite this controversy, most of studies have pointed out a decrease in PA after the extrusion process [43] that was attributed to the thermal degradation of this compound as a consequence of high barrel temperatures [44].

A significant negative effect of extrusion was observed for GABA (Table 3), which was reduced by 26% and 35% for 100% SQF and SCF (formulations #2 and #3), respectively, as compared to that in their corresponding unprocessed flours (Table 2, *p* ≤ 0.05). In contrast, CG extrudates (formulation #1) showed similar GABA values as compared to that in unprocessed CG. A partial breakdown of GABA (between 1.26 and 2.44 times) was also reported by Chalermchaiwat et al. [45] in sprouted brown rice flour after extrusion. GABA degradation may occur at a high barrel temperature (140 °C), but other factors like low feed moisture and high screw speed may negatively affect GABA content in extrudates [45]. In summary, heat and shear forces have been reported as the main factors causing GABA loss in cereal extrudates [46].

Extrusion caused different effects depending on the raw material used. When CG was the only ingredient in the formulation, TSPC and ORAC significantly decreased (17.5 and 18.9%, respectively) after the extrusion process in CG extrudates (*p* < 0.05, Table 3) as compared to those in unprocessed sample (Table 2). Opposite effects were observed for formulations containing exclusively sprouted pseudocereal flours (TSPC increased by 24 and 73%; ORAC increased by 58% and 7%, respectively; test #2 and #3, Table 3 vs. SCF and SQF in Table 2). The existing literature supports the findings of this study, reflecting controversial results regarding the effect of extrusion on TSPC and antioxidant activity. These effects depend on factors including intrinsic chemical composition of raw materials and extrusion processing conditions [47]. While several studies demonstrated that phenolic compounds in cereals are thermally degraded during extrusion, causing a loss of free phenolic content and antioxidant activity [48], other investigations reported an increase of TSPC promoted by the combination of pressurization, shearing and heating-induced hydrolysis of conjugated phenolic compounds [49,50]. For instance, phenolic compounds in coffee parchment flour are liberated from lignin and hemicelluloses owing to the extended extraction effect of the screw under high temperature and pressure, which contributes to the increase in antioxidant activity [50]. Moreover, the generation of Maillard reaction products during extrusion may also contribute to increased ORAC in extrudates [47].

**Figure 2:**
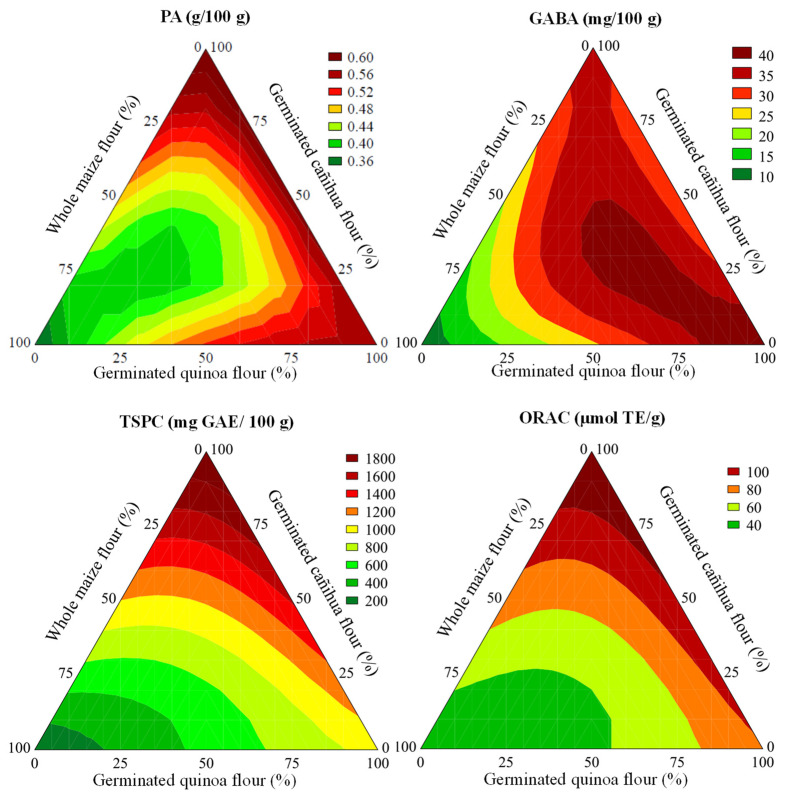
Contour plots of predicted phytic acid (PA), γ-aminobutyric acid (GABA), total soluble phenolic compounds (TSPC) and oxygen radical absorbance capacity (ORAC) in extrudates elaborated with different ratios of CG, SQF and SCF.

The regression model for PA showed three main components of influence for their higher coefficients of regression (Supplementary Material, Table S1). Specifically, PA content was positively affected by the linear ratio of SQF and SCF (β_2_ = +0.54 and β_3_ = +0.60, respectively) in extrudate formulations. On the other hand, there was a negative effect on PA content of extrudates related to the use of ternary blends CG–SQF–SCF (β_123_ = −3.31). These main effects of the type of flour in the extrudate formulations are shown in Figure 2. Extrudates elaborated with high ratios of SQF and SCF flours showed maximum levels for PA, a nondesirable nutritional parameter due to its mineral chelating properties.

A positive linear interaction effect of sprouted pseudocereals’ ratio on the GABA content in extrudates was revealed from regression models (Supplementary Material, Table S1). As shown in Figure 2, GABA enrichment in extrudates was maximum with an almost complete substitution of CG by SQF or binary blends SQF–SCF in which the SQF ratio was prominent in the extrudate formulation. Regarding TSPC and ORAC, there was a similar and clear positive linear effect of the SCF ratio in the extrudate formulation (Supplementary Material, Table S1). Maximum TSPC and ORAC values were reached in formulations elaborated using SCF as the main ingredient (>75%, Figure 2). These observations are in line with the nutritional composition and antioxidant activity of raw materials collected in Table 2.

### 3.3. Effect of CG Replacement by Sprouted Pseudocereal Flours on Physical Properties of Extrudates

**Figure 3:**
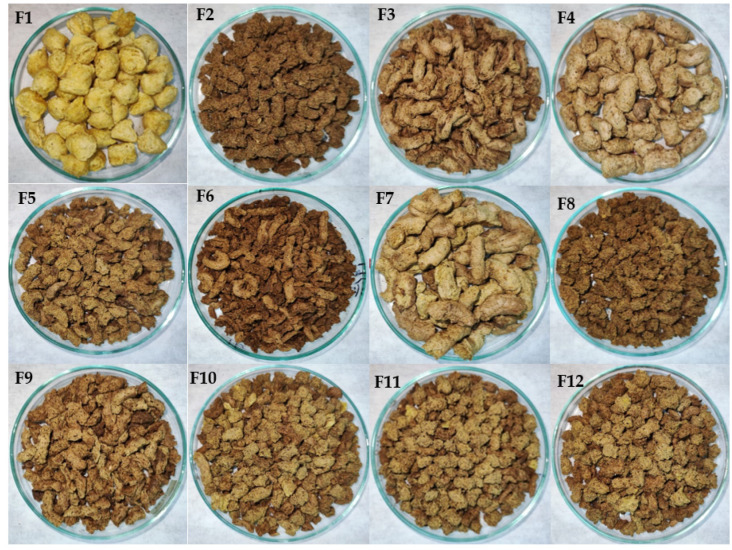
Images of extrudates made of CG (**F1**), SQF (**F2**), SCF (**F3**), binary (**F4**–**F6**) and ternary (**F6**–**F12**) flour blends of the same. Abbreviations: CG, corn grit; SCF, sprouted cañihua flour; SQF, sprouted quinoa flour.

Color and texture are among the quality attributes directly related to the acceptability of extrusion products that are affected by ingredient formulation [29,51]. The images of extrudates produced from single, binary and ternary blends of CG, SQF and SCF are presented in Figure 3. These images were provided for visual comparison and gave a preliminary insight into the physicochemical quality properties of the extrudates such as EI, BD and color.

Table 4 shows a variation in the EI of extrudates that ranged from 0.88 to 2.10 as influenced by formulation (Table 4). These values are in accordance with earlier work showing an EI of 1.33 and 2.49 for extrudates formulated using ratios 50:50 and 70:30 of quinoa and CG, respectively [52]. The mathematical model explained 95.26% of the data, which was considered as highly predictive for presenting the value of F_calc_ 18.37 times higher than F_tab (3;8;0.10)_ (*p* < 0.001, Supplementary Material, Table S1). The highest extruded expansion was observed with CG (β_1_ = 2.04), followed by that with SCF (β_3_ = 0.97, Supplementary Material, Table S1). However, the interaction between CG and SCF had a deleterious effect on the expansion of the material during the thermoplastic extrusion process (β_13_ = −1.79, Supplementary Material, Table S1). The EI is an important factor to be monitored because it affects the extrudates’ density, fragility and crunchiness. SQF had lower starch content (55.89%, Table 2) than common cereals used in extrusion technology did, for instance, corn, containing 71.15% of starch (Table 2). For this reason, the formulations with the greatest replacement of germinated pseudocereal flours present a lower EI, since higher starch and amylopectin content are associated with better EI values [5].

**Figure 4:**
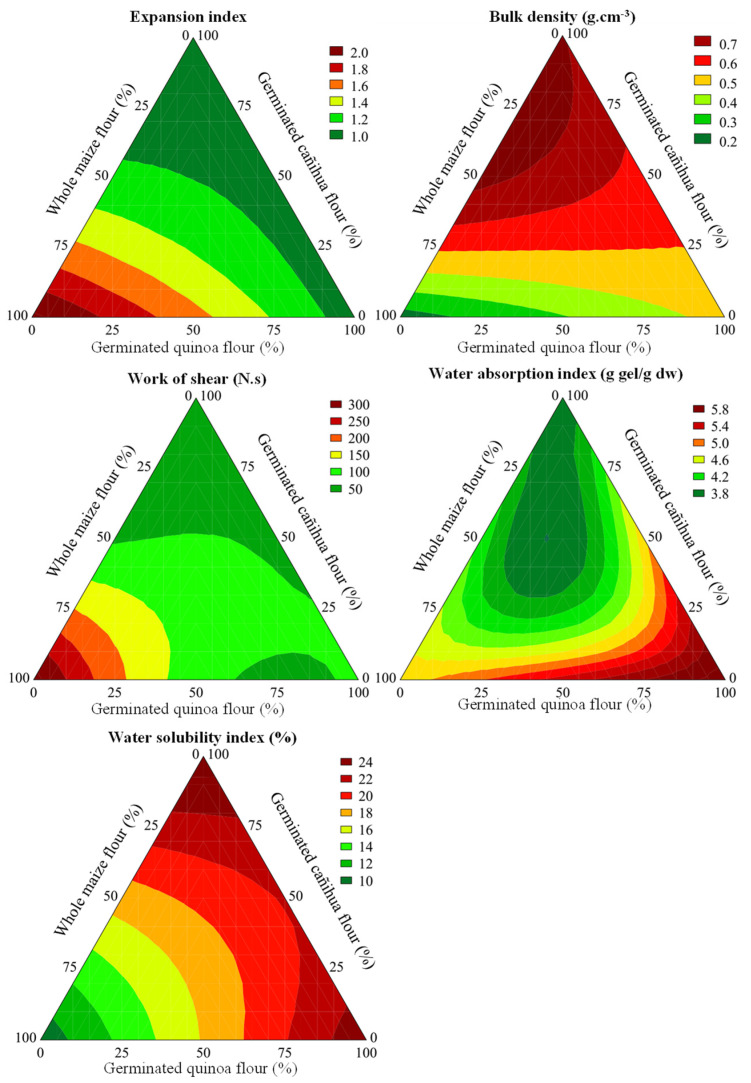
Contour plots of predicted expansion index, bulk density, shear work, water absorption index and water solubility index.

A contour plot (Figure 4) shows that the highest EI was obtained with a higher percentage of CG and lower levels of germinated pseudocereal flours, particularly SQF. During the germination process, the hydrolysis of macronutrients such as starch, proteins and lipids gives rise to low-molecular-weight sugars, amino acids and free fatty acids to provide energy and nitrogen to the grain for the sprouting process. These effects negatively affects the EI due to the lower capacity of food matrix components in sprouts retaining water vapor since the material transposes the die orifice of the extruder and reaches environmental pressure [5]. Taking into account the nutritional composition, SQF had higher starch content than SCF did (Table 2), which explained higher EI in formulations with the highest quinoa ratio (Table 4, Figure 4). Starch content is not the only factor that has influence on the EI, because the proportion between amylose and amylopectin and the levels of intermediate glucans also do [53]. Although high values of EI are required for conventional expanded extrudates, there are scientific reports by some researchers that consumers accept denser extruded products, correlating them to healthier foods from a nutritional point of view, although technological quality could be inferior [54].

The BD of experimental extrudates varied from 0.22 to 0.73 g/cm^3^ (Table 4). These values were similar to those found in the literature (0.199 and 0.427 g/cm^3^) for extrudates formulated with sweet potato, quinoa and tarwi flours. The mathematic model was considered predictive (F_calc_/F_tab (3;8;0.10)_ = 3.69, Supplementary Material, Table S1) and explained 80.16% of data variability (*p* = 0.003). BD is inversely related to EI, as products with a better EI show lower BD and more porous structures. High values of specific mechanical energy in the extrusion process result in a positive effect on expansion, a growth of gas bubbles and a less dense and more brittle final texture [55]. BD expresses an imperative relationship with its ability to sink or float when added to any liquid such as water, milk, yogurt or dairy drink [56]. According to Figure 4, the lowest BD values were obtained with the highest levels of CG (β_1_ = 0.15, Supplementary Material, Table S1) and lower germinated pseudocereal flour levels (β_2_ = 0.15, Supplementary Material, Table S1) for a higher EI. Additionally, binary effects between CG and SCF resulted in a denser product (β_13_ = 1.21). The enzymatic hydrolysis that takes place during grain germination explains these findings, as the disintegration of food matrix components in sprouts is related to the loss of EI.

The results of the instrumental texture (SF and WF) of the experimental trials are presented in Table 4. From the physical point of view, texture is related to the force required to produce deformation and the force required to compress a substance between interdental spaces. SF values ranged between 10.16 and 58.49 N. Although 81.86% of the results were explained by the mathematical model, the prediction could not be guaranteed since the ratio between F_calc_/F_tab (6;5;0.10)_ was only 1.11. For WS, the data ranged between 14.11 and 331.66 N·s, and 95.15% of this variability was explained by the predictive mathematical model (F_calc_/F_tab (5;6;0.10)_ = 7.58, Supplementary Material, Table S1). It was noticed that the negative effect of germinated pseudocereal flours on the EI and BD was minimized for WS, since the interactions between CG and SQF (β_12_ = −467) and CG and SCF (β_13_ = −0.500, Supplementary Material, Table S1) showed the best performances, in accordance with the contour plot (Figure 4). Probably, a higher CG ratio resulted in a large width of the cell wall for gas bubbles (as observed for WS for β_1_ = 318, Supplementary Material, Table S1). The presence of lower molar weight for digestible carbohydrates (starch) and proteins in sprouted flours could decrease the adverse effects, resulting in a better extrudate structure. The highest WS values come from irregular thickness of the cell wall of the air bubbles, requiring greater forces to break the structure of each bubble during the extruded cutting step in the texture analysis. With the germination process and the action of endogenous enzymes there was a reduction in the macromolecular structure of the starch, resulting in lower medium viscosities during the extrusion process, which may have promoted thinner walls for air cells, thus decreasing the total force required over time for snack disintegration [57].

Table 4 shows a clear variation of WAI (3.07–5.96 g of gel/g sample) and WSI (8.44–24.85) as influenced by the formulation of extrudates, in agreement with earlier work [52]. Predictive mathematic models for WAI (F_calc_/F_tab (3;8;0.10)_ = 5.51) and WSI (F_calc_/F_tab (3;8;0.10)_ =12.95) explained 85.79% and 93.41% of data variability, respectively (Supplementary Material, Table S1). The raw materials applied in the extrusion process showed WAI values (g of gel/g of sample in dw) of 2.38 ± 0.04 for CG, 3.94 ± 0.27 for SQF, and 3.96 ± 0.11 for SCF, where SCF showed the highest value and CG had the lowest water absorption capacity (*p* < 0.05). For WSI, the highest value was found for SCF (14.89 ± 0.43%), followed by that for SQF (10.60 ± 0.59%) and the lowest one for CG (4.09 ± 0.17%) (*p* < 0.05). The WAI is an indicator of the amount of water absorbed by the starch in dispersion, which indicates the degree of gelatinization of the food. In snack production, WAI values should be kept to minimal levels, whereas high WSI values are appreciated. These parameters indicate a good sensory quality for extrudates, as the consumer, by chewing the product with a few bites, expects the snack to dissolve in saliva without forming a gummy texture for mouthfeel. To achieve these characteristics, thermodextrinization is necessary in the raw material. Starch degradation through mechanical work in the presence of high temperatures and low moisture results in the formation of low-molar-weight dextrins [58]. WAI values were negatively influenced by the ratio of SCF (β_3_ = 3.59, Supplementary Material, Table S1) and the interaction of ternary blends of ingredients (β_123_ = −16.27, Supplementary Material, Table S1). On the other hand, the largest WSI was achieved with higher ratios of SCF (β_3_ = 25.22, Supplementary Material, Table S1) and SQF (β_2_ = 23.59, Supplementary Material, Table S1). Enzymatic hydrolysis of macromolecules induced by germination that leads to a decrease of the molecular weight of starch granules may explain the reduced WAI and increased WSI of extrudates containing higher ratios of sprouted pseudocereal flours [14].

According to the general knowledge applied to the production of expanded extruded (snacks), higher EI and WSI values and lower levels of BD, WAI and instrumental texture are required. However, these characteristics are reached using refined flour (without the presence of germ and dietary fiber from the pericarp portion) and that have not been subjected to technological processes such as germination or pre-gelatinization. These snacks generally have a high glycemic index and are highly caloric due to the rapid conversion of starch into glucose and the use of a lipid fraction to impregnate aromas and salt after the drying process of the snacks. The flours obtained from germinated grains promoted the reduction of the major technological properties of snacks but resulted in improved nutritional and healthy effects. The partial mixture of the germinated flours with whole cornmeal improved the physicochemical properties of the extrudates as well as enhanced the health attributes of the final product.

Instrumental color parameters L*, a* and b* are presented in Table 4. The luminosity values (L*) of the trials ranged from 34.17 to 61.23 and were 98.61% explained by the mathematical model, which was considered predictive (F_calc_/F_tab (4;7;0.10)_ = 42.08, *p* <0.001; Supplementary Material, Table S1). The highest values of L* were obtained at the highest proportion of CG (β_1_ = 61.26, Supplementary Material, Table S1). On the other hand, the highest proportion of SQF and SCF showed the lowest luminosity values (β_2_ = 40.78 and β_3_ =35.72). Likewise, binary mixtures of CG–SCF (β_12_ = −27.49, Supplementary Material, Table S1) and SQF–SCF (β_23_ = −17.15, Supplementary Material, Table S1) showed negative effects on the luminosity of the samples. In this sense, it was inferred that incorporation of SQF and SCF in the formulation was responsible for the darkening of the extrudates. This result is advantageous because consumers have a sensory memory that wholegrain cereal and pseudocereal products that are considered healthy have a darker color [59]. Muñoz-Pabon and collaborators [60] extruded a hyper-protein quinoa flour mix with a corn flour, rice flour and cassava starch in a single screw extruder at a die temperature of 140 °C and also found a decrease in luminosity (L* from 54.03 with 25% quinoa flour to 51.49 with 37% quinoa flour and 62.68 without quinoa flour). The authors related this effect to the raw material color, the browning reaction, lower expansion index and higher bulk density.

Parameter a* of the instrumental color of the trials ranged from 5.99 to 10.73 (Table 4). The mathematical model (F_calc_/F_tab (5;6;0.10)_ = 8.42; *p* = 0.001, Supplementary Material, Table S1) could explain 95.62% of experimental results. The largest shade of a* was obtained with the use of the ternary component CG–SQF–SCF (β_123_ = 40.49; Supplementary Material, Table S1).

**Figure 5:**
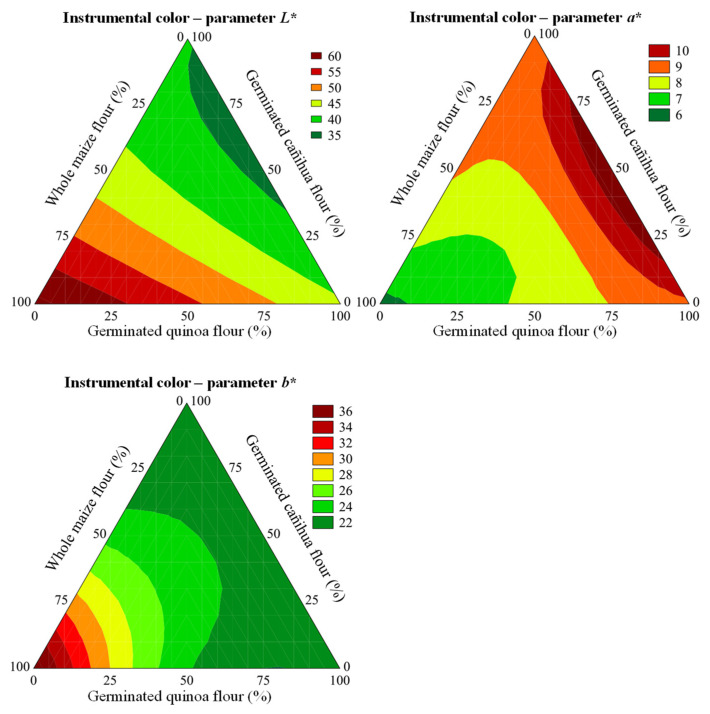
Contour plots predicted for instrumental color parameters (L*, a*, b*).

Parameter b* showed values between 20.52 and 37.65 (Table 4). The mathematical model (F_calc_/F_tab (5;6;0.10)_ = 19.67; *p* < 0.001; Supplementary Material, Table S1) explained 98.08% of the experimental results. Binary components CG–SQF (β_12_ = −27.49; Supplementary Material, Table S1) and SQF–SCF (β_13_ = 22.71; Supplementary Material, Table S1) promoted a decrease in parameter b*. A greater b* indicates a yellowish color, characteristic of the presence of carotenoids found in cereals and pseudocereals, mainly from lutein, zeaxanthin β-cryptoxanthin and β-carotene found in corn, quinoa [61] and cañihua [62]. These carotenoids are thermally degraded during the thermoplastic extrusion process, resulting in a partial loss of b* [63]. All observed color modifications (lower values of L* and higher values of a* and b*) are related to the natural color of the added ingredients, but also typical of the generation of brown pigments due to the Maillard reaction [5]. In this sense, higher ratios of SQF and SCF resulted in extrudates with lower L* and b* (Figure 5) and increased a* related to a brownish color, most likely due to the presence of higher amounts of precursors of the Maillard reaction such as reducing sugars and free amino acids (Figure 3).

### 3.4. Optimal Formulation Improved the Nutritional Quality, Texture and Color of Corn-Sprouted Pseudocereal Flour Extrudates

Multiresponse optimization using a desirability function was performed to identify the overall optimal formula using SQF and SCF as replacements of CG. Each variable was assigned to a level of importance (where 1 and 5 were low and high level of importance, respectively) in accordance with the desired objective: high supplementation ratio of SQF and SCF to maximize GABA, TSPC, ORAC, IE, WSI and a* and minimize PA, BD, WS and b*. Based on these established criteria, two optimal formulations of extrudates were selected: Optimal formulation 1 (OPM1: 0% CG, 12% SQF and 86% SCF) and Optimal formulation 2 (OPM2: 24% CG, 17% SQF, 59% SCF) (Table 5), and the nutritional composition of the optimized experimental extrudates was compared (Table 6). The experimental value for the validation of each of the responses was compared with those predicted by the mathematical model. The tests were performed in triplicate, and the values are also listed in Table 5. All mathematical models were validated since the relative deviation was between 5% and 20% for most of the response variables.

The comparative analysis of the nutritional composition of the control (100% CG) and extrudates elaborated with the optimized formulations based of binary SQF–SCF (OPM1) and ternary CG–SQF–SCF (OPM2) blends is presented in Table 6. Overall, the extrudates containing sprouted pseudocereal flours had a greater nutritional value than CG extrudates did (*p* < 0.05). The optimized formulations showed half of the starch content of the CG extrudates. This means that optimized extrudates containing sprouted pseudocereals had a lower glycemic load, which is a desirable feature to prevent metabolic disorders [25].

As compared with that of pure corn extrudate, OPM1 and OPM2 had about three times more TDF with an SDF–IDF ratio of 1:3 and 1:2 (respectively), a double amount of protein and slightly higher content of fat (1.7- and 1.2-fold for OPM1 and OPM2, respectively). A new nutrition facts panel declares 28 g/d as the daily value intake of fiber, based on a 2000-calorie diet [64]. Therefore, OPM1 and OPM2 extrudates could be excellent or good sources of dietary fiber, as their values accounted for 33% and 27%, respectively.

Similar and slightly reduced PA content was observed for OPM1 and OPM2, respectively, as compared to that of the control. This effect could result from sprouting, which activates endogenous phytase [65]. As for GABA and TSPC, the optimized extrudates were better sources of these bioactive compounds with levels between five and eight times higher as compared to those of the control extrudates. Although in Europe GABA does not have health claims by the EFSA, there is scientific evidence supporting beneficial physiological effects of GABA supplements, including modulation of glucose homeostasis, blood pressure modulation and pancreatic and immune functions [66]. In a recent study, de Bie et al. [66] suggested that GABA bioavailability is not reduced by the food matrix, and food products containing GABA could potentially induce health effects similar to those claimed for GABA supplements. Regular dietary intake of polyphenols, approximately 1–2 g per day, has been associated with chronic disease prevention [39]. Although there are no official recommendations for phenolic compounds intake, a serving of 100 g of OPM1 and OPM2 could reach the reported mean daily intake (1 and 1.2 g/d) [40].

Similarly, extrudates containing sprouted pseudocereals were characterized by 5- and 3.5-fold greater ORAC values than that of corn extrudates.

The incorporation of 23% CG in the extrudate formulation (OPM2) resulted in a reduction of the overall nutritional value as compared to that of OPM1 (for instance, higher starch content and lower protein, TDF, protein, fat, ash, PA, TSPC and ORAC levels).

### 3.5. Gastric and Intestinal Digestates of Optimized Extrudates Had Greater PA, GABA, TSPC and ORAC

PA content was reduced during digestion, reaching the lowest amounts at the end of intestinal digestion (Table 6, *p* < 0.05). PA degradation might occur in the stomach due to activation of endogenous phytases in the food matrix in the acidic environment of the stomach as a consequence of lower pH or in the colon due to the fermentation of dietary fibers by specific microbiota species with high phytase activity [55].

GABA concentration was not influenced by gastric and intestinal digestion, as similar values were observed in digestates as compared to those in undigested extrudates (Table 6). Nonetheless, the amounts of bioaccessible GABA at the end of intestinal digestion were greater in the control extrudates as compared to that in the undigested sample (Table 6). Similar observations were reported earlier by Dala-Paula et al. [57], who indicated that GABA content increased after the in vitro digestion of chocolate due to digestive enzymes, which agreed with results herein. The resistance of GABA to the gastric environment (pH 1.2) and intestinal conditions (pH 7) was also reported for GABA-rich yoghurt [56].

TSPCs increased at the end of gastric digestion for all extrudates, and the highest amounts of bioaccessible TSPC were detected in OPM1, followed by those in OPM2 and control. The effect of intestinal digestion varied in control and optimized extrudates. This is in agreement with previous work of Dust et al. [67], who concluded that the effects of extrusion on hydrolytic and fermentative digestion are influenced by the unique chemical characteristics of individual food matrices. In the present work, bioaccessibility of TSPC decreased in the intestine for OPM1 and OPM2 as compared to that in the gastric phase, although TSPC amounts were higher than those found for undigested extrudates. Unlike optimized extrudates, the release of TSPC continued until the end of intestinal digestion in corn extrudates (Table 6), although the concentration of bioaccessible polyphenols did not reach those reported for OPM1 and OPM2. Similar results were reported for apple pomace extrudates [68]. Changes in phenolic compounds’ bioaccessibility are due to an interplay of the stability of the individual polyphenols in various gastric and intestinal fluids and the increased release of polyphenols due to digestion of the matrix as well as binding of polyphenols to exposed binding sites as a result of digestion of matrix components [68]. Polyphenols are stable under acidic conditions but are prone to degradation under alkali conditions. In the gastric phase, glycosidic bonds can be broken under acidic conditions to release bound phenols, which explain the higher TSPC in gastric digestates for all extrudates.

ORAC increased gradually from the gastric to the intestinal phases of digestion for OPM1 and OPM2. These results were similar to those reported in a previous study of the changes of antioxidant activity during digestion of apple pomace extrudates [68]. Many factors can account for this increase in antioxidant activity, such as contribution from the phenolic compounds themselves due to their release and conversion reactions.

## 4. Conclusions

SQF and SCF had less starch and more protein, dietary fiber, lipids and minerals than CG did, giving these alternative flours a promising opportunity in the production of extrudates. The extrusion process did not influence PA but caused different effects on GABA, TSPC and ORAC depending on the raw material used. Extrusion reduced GABA content in extrudates formulated exclusively with SCF or SQF, while no effects were observed for CG extrudates. When CG was the only ingredient in the formulation, TSPC and ORAC significantly decreased after the extrusion process, but opposite effects were observed for formulations containing 100% sprouted pseudocereal flours.

The optimization of flour blends using a simplex centroid mixture design and a further multiresponse desirability method allowed obtaining extrudates with improved nutritional quality and physicochemical parameters related to color and texture closer to desirable values. Physicochemical properties, such as EI and WSI, were improved with the addition of CG to the sprouted pseudocereals. From a nutritional point of view, the optimized extrudates were characterized by lower starch content and improved amounts of dietary fiber, protein, fat, minerals, bioactive compounds (GABA, TSPC) and antioxidant activity. Further, in the digestion process, optimized extrudates gave rise to higher amounts of bioaccessible bioactives and antioxidant activity at the stomach and intestine level. All these results encourage manufacturers to more widely use SQF and SCF to create and promote healthier versions of cereal-based products, accelerating the transition to healthy and sustainable food systems.

## Figures and Tables

**Table 1:** Simplex centroid mixture design including 12 experimental flour formulations.

Test	Flour Blend Ratio		
	CG	SQF	SCF
**1**	100	0	0
**2**	0	100	0
**3**	0	0	100
**4**	50	50	0
**5**	50	0	50
**6**	0	50	50
**7**	66.67	16.67	16.67
**8**	16.67	66.67	16.67
**9**	16.67	16.67	66.67
**10**	33.33	33.33	33.33
**11**	33.33	33.33	33.33
**12**	33.33	33.33	33.33

Abbreviations: CG, corn grit; SCF, sprouted cañihua flour; SQF, sprouted quinoa flour.

**Table 2:** Nutritional composition (expressed as g/100 g dry weight, dw), TSPC (mg GAE/100 g), GABA (mg/100 g) and antioxidant activity (expressed in μmol TE/g) of raw materials.

Parameters	CG	SQF	SCF
Moisture	11.12 ± 0.74 ^b^	8.94 ± 0.26 ^a^	12.03 ± 0.13 ^b^
Starch	71.15 ± 0.12 ^c^	55.84± 0.52 ^b^	41.21 ± 1.47 ^a^
TDF	5.06 ± 0.74 ^a^	18.84 ± 1.20 ^b^	22.16 ± 0.83 ^c^
IDF	1.19 ± 0.33 ^a^	12.34 ± 1.06 ^b^	15.28 ± 0.95 ^c^
SDF	3.87 ± 1.08 ^a^	6.14 ± 0.14 ^b^	6.88 ± 0.12 ^b^
Protein	7.22 ± 0.05 ^a^	23.36 ± 4.38 ^b^	19.11 ± 0.27 ^b^
Fat	3.95 ± 0.02 ^a^	6.55 ± 0.11 ^b^	6.23 ± 0.25 ^b^
Ash	0.85 ± 0.09 ^a^	3.66 ± 0.11 ^c^	2.68 ± 0.05 ^b^
PA	0.37 ± 0.01 ^a^	0.58 ± 0.01 ^b^	0.56 ± 0.03 ^b^
GABA	4.02 ± 0.03 ^a^	55.43 ± 2.37 ^b^	51.55 ± 4.35 ^b^
TSPC	171.11 ± 8.95 ^a^	525.50 ± 38.14 ^b^	1545.09 ± 111.33 ^c^
ORAC	23.47 ± 1.32 ^a^	45.30 ± 3.96 ^b^	114.92 ± 14.17 ^c^

Data are means ± standard deviation of three replicates (*n* = 3). Different letters denote statistical differences among raw materials (ANOVA, Bonferroni *post hoc* test, *p* ≤ 0.05). Abbreviations: CG: corn grit; GABA, γ-aminobutyric acid; GAE, gallic acid equivalents; IDF: insoluble dietary fiber; ORAC, oxygen radical absorbance capacity; PA, phytic acid; SCF; sprouted cañihua flour; SDF, soluble dietary fiber; SQF, sprouted quinoa flour; TE, Trolox equivalents; TDF, total dietary fiber; TSPC, total soluble phenolic compounds.

**Table 3:** PA, GABA, TSPC and ORAC in extrudates elaborated with different ratios of CG, SQF and SCF.

Test	CG (x_1_)	SQF (x_2_)	SCF (x_3_)	PA (g/100 g)	GABA (mg/100 g)	TSPC (mg GAE/100 g)	ORAC (µmol TE/g)
1	100	0	0	0.36 ± 0.03	5.19 ± 0.18	141.1 ± 11.2	19.10 ± 1.48
2	0	100	0	0.53 ± 0.04	41.02 ± 0.46	910.4 ± 60.4	71.52 ± 3.51
3	0	0	100	0.62 ± 0.01	33.67 ± 0.62	1917.7 ± 149.1	122.67 ± 2.50
4	50	50	0	0.50 ± 0.01	25.56 ± 1.16	399.0 ± 10.3	33.88 ± 2.65
5	50	0	50	0.46 ± 0.01	22.81 ± 0.36	908.9 ± 57.3	75.27 ± 1.65
6	0	50	50	0.57 ± 0.01	27.13 ± 0.45	1320.97± 99.2	103.84 ± 3.43
7	66.67	16.67	16.67	0.35 ± 0.03	22.83 ± 1.03	265.6 ± 73.5	32.60 ± 0.80
8	16.67	66.67	16.67	0.52 ± 0.00	34.72 ± 2.94	839.56 ± 43.7	70.38 ± 2.86
9	16.67	16.67	66.67	0.47 ± 0.02	28.40 ± 0.12	1592.6 ± 139.5	81.79 ± 3.70
10	33.33	33.33	33.33	0.42 ± 0.05	36.88 ± 0.31	713.88 ± 66.1	52.00 ± 0.65
11	33.33	33.33	33.33	0.41 ± 0.03	36.66 ± 0.31	698.1 ± 35.3	48.19 ± 0.05
12	33.33	33.33	33.33	0.38 ± 0.03	37.10 ± 0.31	660.4 ± 26.7	44.99 ± 0.84

Data are means ± standard deviation of three replicates (*n* = 3). Abbreviations: CG, corn grit; GABA, γ-aminobutyric acid; GAE, gallic acid equivalents; ORAC, oxygen radical absorbance capacity; PA, phytic acid; SCF, sprouted cañihua flour; SQF, sprouted quinoa flour; TE, Trolox equivalents; TSPC, total soluble phenolic compounds.

**Table 4:** Physical properties of extrudates made with CG, SQF and SCF used in single, binary and ternary blends.

Tests	CG (X_1_)	SQF (X_2_)	SCF (X_3_)	EI	BD (g/cm^3^)	SF (N)	SW (N·s)	WAI (g gel/g dw)	WSI (%)	L*	a*	b*
1	100	0	0	2.10 ± 0.06	0.22 ± 0.02	32.48 ± 3.17	331.66 ± 27.53	4.48 ± 0.18	8.44 ± 0.16	61.23 ± 1.41	5.99 ± 0.21	37.65 ± 0.55
2	0	100	0	1.00 ± 0.08	0.46 ± 0.07	21.24 ± 2.56	67.05 ± 10.24	5.96 ± 0.14	24.75 ± 1.46	41.59 ± 0.29	8.80 ± 0.18	21.08 ± 0.40
3	0	0	100	0.94 ± 0.02	0.73 ± 0.09	10.16 ± 1.25	14.11 ± 2.25	4.06 ± 0.01	24.85 ± 0.09	34.89 ± 1.07	8.03 ± 0.20	20.63 ± 0.75
4	50	50	0	1.41 ± 0.07	0.19 ± 0.02	15.46 ± 1.69	88.86 ± 13.24	5.30 ± 0.10	15.38 ± 0.07	51.61 ± 0.56	6.96 ± 0.09	22.67 ± 0.52
5	50	0	50	1.04 ± 0.09	0.72 ± 0.10	37.36 ± 4.00	48.68 ± 5.96	4.01 ± 0.17	17.63 ± 0.82	41.78 ± 1.27	8.14 ± 0.19	23.50 ± 0.88
6	0	50	50	0.88 ± 0.11	0.60 ± 0.05	26.92 ± 5.78	27.93 ± 5.43	4.55 ± 0.11	21.78 ± 0.74	34.17 ± 0.46	10.73 ± 0.37	20.52 ± 0.42
7	66.67	16.67	16.67	1.47 ± 0.04	0.32 ± 0.05	23.31 ± 2.59	94.32 ± 16.85	4.77 ± 0.23	15.65 ± 1.65	50.35 ± 0.97	6.43 ± 0.08	25.73 ± 0.28
8	16.67	66.67	16.67	1.00 ± 0.05	0.40 ± 0.05	22.62 ± 1.89	42.80 ± 27.53	4.89 ± 0.07	17.83 ± 0.33	38.18 ± 0.95	9.22 ± 0.10	21.36 ± 0.43
9	16.67	16.67	66.67	1.11 ± 0.09	0.56 ± 0.08	49.96 ± 6.97	51.10 ± 5.73	3.07 ± 0.47	20.27 ± 0.19	36.92 ± 1.15	9.13 ± 0.16	22.52 ± 0.50
10	33.33	33.33	33.33	1.09 ± 0.03	0.66 ± 0.07	58.41 ± 8.13	78.34 ± 10.56	3.76 ± 0.01	16.55 ± 0.12	41.56 ± 0.51	7.46 ± 0.25	22.82 ± 0.81
11	33.33	33.33	33.33	1.05 ± 0.05	0.66 ± 0.07	57.52 ± 5.41	81.26 ± 8.72	3.76 ± 0.08	16.42 ± 0.22	41.70 ± 0.36	7.55 ± 0.12	23.17 ± 0.36
12	33.33	33.33	33.33	1.05 ± 0.07	0.67 ± 0.07	57.30 ± 7.35	76.93 ± 7.51	3.74 ± 0.06	16.50 ± 0.15	40.89 ± 0.35	7.73 ± 0.08	22.53 ± 0.32

Data are means ± standard deviation of three replicates. Abbreviations: BD, bulk density; CG, corn grits; EI, expansion index; SCF, sprouted cañihua flour; SQF, sprouted quinoa flour; SF, shear force; SW, shear work; WAI, water absorption index; WSI, water solubility index.

**Table 5:** Parameters selected for the desirability optimization method, optimal extrudate formulations and predicted values for each response variable.

Independent Variables	Criteria	Lower Level	Upper Level	Importance Level	Solution 1 (OPM1)		Solution 2 (OPM2)	
CG (%)	in range	0	1	3	0		24	
SQF (%)	in range	0	1	3	14		17	
SCF (%)	in range	0	1	3	86		59	
**Response Variables**	**Criteria**	**Lower Level**	**Upper Level**	**Importance level**	**Predicted Values**	**Experimental Values**	**Predicted Values**	**Experimental Values**
PA	minimize	0.35	0.62	5	0.54	0.33	0.47	0.23
GABA	maximize	5.19	41.02	5	29.48	37.10	30.80	41.81
TPC	maximize	196.30	1917.75	5	1817.17	1511.46	1168.84	972.7
ORAC	maximize	19.10	122.67	5	115.78	101.79	73.78	69.62
EI	maximize	0.88	2.10	1	0.96	0.88	0.96	1.03
BD	minimize	0.19	0.73	1	0.67	0.71	0.70	0.72
SW	minimize	14.11	331.66	5	25.78	33.18	39.31	88.68
L*	in range	47.41	76.30	5	50.93	39.16	53.61	39.46
a*	maximize	4.87	8.01	5	7.41	7.00	7.39	7.55
b*	minimize	14.06	36.40	5	17.12	17.94	19.46	19.99
WAI	minimize	3.07	5.96	5	3.91	4.18	3.61	3.41
WSI	maximize	8.44	24.85	5	22.95	22.71	19.27	17.84
***Desirability***					***0.7420***	***-***	***0.6451***	***-***

Abbreviations: BD, bulk density; CG, corn grits; EI, expansion index; SCF, sprouted cañihua flour; GABA, γ-aminobutyric acid; ORAC, oxygen radical absorbance capacity; OPM1: optimal formulation 1; OPM2: optimal formulation 2; PA, phytic acid; SQF, sprouted quinoa flour; SF, shear force; SW, shear work; TSPC, total soluble phenolic compounds; WAI, water absorption index; WSI, water solubility index.

**Table 6:** Nutritional composition of corn (control) and optimized extrudates elaborated with selected formulations 1 (OPM1) and 2 (OPM2). Bioaccessible (soluble) amounts of PA, GABA, TSPC and ORAC during in vitro simulated gastrointestinal digestion.

Parameters	Control	OPM1	OPM2
***Undigested extrudates (time endpoint = 0 min)***			
Starch (g/100 g dw)	71.14 ± 0.12 ^c^	33.93 ± 0.20 ^a^	44.05 ± 1.39 ^b^
TDF (g/100 g dw)	9.88 ± 0.14 ^a^	32.95 ± 1.71 ^c^	26.69 ± 2.41 ^b^
IDF (g/100 g dw)	6.87 ± 0.18 ^a^	24.35 ± 2.41 ^c^	18.04 ± 0.46 ^b^
SDF (g/100 g dw)	3.02 ± 0.04 ^a^	8.59 ± 0.69 ^b^	8.65 ± 2.87 ^b^
Protein (g/100 g dw)	7.22 ± 0.05 ^a^	16.28 ± 0.19 ^c^	13.73 ± 0.50 ^b^
Fat (g/100 g dw)	3.95 ± 0.02 ^a^	6.69 ± 0.02 ^c^	4.81 ± 0.24 ^b^
Ash (g/100 g dw)	0.85 ± 0.09 ^a^	3.64 ± 1.23 ^c^	1.82 ± 0.10 ^b^
PA (g/100 g dw)	0.36 ± 0.03 ^b,C^	0.33 ± 0.03 ^b,C^	0.23 ± 0.07 ^a,B^
GABA (mg/100 g)	5.19 ± 0.18 ^a,B^	37.10 ± 0.40 ^b,A^	41.81 ± 0.53 ^c,A^
TSPC (mg GAE/100 g)	196.3 ± 5.0 ^a,A^	1511.5 ± 24.7 ^c,A^	973.3 ± 23.0 ^b,A^
ORAC (μmol TE/g)	19.85 ± 2.60 ^a,A^	101.79 ± 1.53 ^c,A^	69.62 ± 1.17 ^b,A^
***Gastric digestates (time endpoint = 120 min)***			
PA (g/100 g dw)	0.19 ± 0.03 ^a,B^	0.17 ± 0.01 ^a,B^	0.21 ± 0.04 ^a,B^
GABA (mg/100 g)	2.13 ± 0.03 ^a,A^	36.88 ± 2.63 ^b,A^	42.72 ± 3.60 ^b,A^
TSPC (mg GAE/100 g)	270.5 ± 18.9 ^a,B^	2493.9 ± 59.7 ^c,C^	1816.2 ± 98.7 ^b,C^
ORAC (μmol TE/g)	57.11 ± 2.42 ^a,B^	118.09 ± 6.28 ^c,B^	82.82 ± 11.51 ^b,B^
***Intestinal digestates (time endpoint = 240 min)***			
PA (g/100 g dw)	0.08 ± 0.03 ^a,A^	0.14 ± 0.10 ^b,A^	0.17 ± 0.01 ^b,A^
GABA (mg/100 g)	11.32 ± 0.38 ^a,C^	34.34 ± 1.60 ^b,A^	38.90 ± 0.47 ^b,A^
TSPC (mg GAE/100 g)	821.3 ± 49.8 ^a,C^	1921.9 ± 278.11 ^c,B^	1248.1 ± 27.4 ^b,B^
ORAC (μmol TE/g)	205.82 ± 25.61 ^a,C^	282.37 ± 10.57 ^b,C^	233.42 ± 7.14 ^a,C^

Data are means ± standard deviation of three replicates. Different lowercase letters denote statistical differences among mean values within a row (ANOVA, Bonferroni post hoc test, *p* ≤ 0.05). Different uppercase letters denote statistical differences among undigested, gastric and intestinal digestates (ANOVA, Bonferroni post hoc test, *p* ≤ 0.05). Abbreviations: dw, dry weight; GABA, γ-aminobutyric acid; GAE, gallic acid equivalents; IDF, insoluble dietary fiber; ORAC, oxygen radical absorbance capacity; PA, phytic acid; SDF, soluble dietary fiber; TDF, total dietary fiber; TE, Trolox equivalents; TSPC, total soluble phenolic compounds.

## Data Availability

The data presented in this study are available in this article or supplementary materials.

## Supplementary Materials

The following supporting information can be downloaded at: https://www.mdpi.com/article/10.3390/foods11203259/s1, Table S1: Predictive regression models describing the relationships between the nutritional and bioactive attributes of extrudates with corn-sprouted pseudocereal flour blends.
