# Supplementary material for: Andean Sprouted Pseudocereals to Produce Healthier Extrudates: Impact in Nutritional and Physicochemical Properties
Source: Foods. 2022 Oct 18;11(20):3259. doi: 10.3390/foods11203259 (PMC9601839; doi:10.3390/foods11203259)
Supplement: Supplementary file 1 [file foods-11-03259-s001.zip › foods-1955852-supplementary.pdf]

Table S1. Predictive regression models describing the relationships between the nutritional and bioactive attributes of extrudates with corn-sprouted pseudocereal flour blends.

Dependent Variables	Mathematical Models	R^2(Pred)	*p*-value
PA	$0.34x_1 + 0.54x_2 + 0.60x_3 + 0.25x_1x_2 - 3.31x_1x_2x_3$	93.95	<0.001
GABA	$7.07x_1 + 41.51x_2 + 35.59x_3 - 46.37x_2x_3 + 371.61x_1x_2x_3$	96.92	<0.001
TSPC	$26x_1 + 878x_2 + 1970x_3 - 6007x_1x_2x_3$	95.91	<0.001
ORAC	$20.69x_1 + 77.76x_2 + 118.91x_3 - 50.84x_1x_2 - 469.18x_1x_2x_3$	97.27	<0.001
Expansion index	$2.04x_1 + 0.90x_2 + 0.97x_3 - 1.79x_1x_3$	95.26	<0.001
Bulk density	$0.15x_1 + 0.43x_2 + 0.71x_3 + 1.21x_1x_3$	80.16	0.003
Shear work	$318x_1 + 62x_2 + 20x_3 - 467x_1x_2 - 500x_1x_3 + 1250x_1x_2x_3$	95.15	<0.001
Water absorption index	$4.69x_1 + 5.90x_2 + 3.59x_3 - 26.27x_1x_2x_3$	85.79	<0.001
Water solubility index	$8.73x_1 + 23.59x_2 + 25.22x_3 - 16.90x_2x_3$	93.41	<0.001
Instrumental color – parameter *L**	$61.26x_1 + 40.78x_2 + 35.32x_3 - 24.32x_1x_3 - 17.15x_2x_3$	98.61	<0.001
Instrumental color – parameter *a**	$5.72x_1 + 8.81x_2 + 8.13x_3 + 4.76x_1x_3 + 10.01x_2x_3 - 40.49x_1x_2x_3$	95.62	<0.001
Instrumental color – parameter *b**	$37.16x_1 + 21.14x_2 + 20.98x_3 - 27.49x_1x_2 - 22.71x_1x_3 + 53.31x_1x_2x_3$	98.08	<0.001

Regression models include only significant terms (ANOVA, $p \leq 0.05$). Independent variables in de equation include ratio of corn grits (x_1); ratio of sprouted quinoa flour (x_2) and ratio of sprouted cañihua flour (x_3); Abbreviations: GABA, γ-aminobutyric acid; ORAC, oxygen radical absorbance capacity; PA, phytic acid; TSPC, total soluble phenolic compounds.
